# Multigenic Delineation of Lower Jaw Deformity in Triploid Atlantic Salmon (*Salmo salar* L.)

**DOI:** 10.1371/journal.pone.0168454

**Published:** 2016-12-15

**Authors:** Gianluca Amoroso, Tomer Ventura, Jennifer M. Cobcroft, Mark B. Adams, Abigail Elizur, Chris G. Carter

**Affiliations:** 1 Institute for Marine and Antarctic Studies (IMAS), University of Tasmania, Private Bag 49, Hobart, Tasmania, Australia; 2 Genecology Research Centre, School of Science and Engineering, University of the Sunshine Coast, Locked Bag 4, Maroochydore DC, Queensland, Australia; Xiamen University, CHINA

## Abstract

Lower jaw deformity (LJD) is a skeletal anomaly affecting farmed triploid Atlantic salmon (*Salmo salar* L.) which leads to considerable economic losses for industry and has animal welfare implications. The present study employed transcriptome analysis in parallel with real-time qPCR techniques to characterise for the first time the LJD condition in triploid Atlantic salmon juveniles using two independent sample sets: experimentally-sourced salmon (60 g) and commercially produced salmon (100 g). A total of eleven genes, some detected/identified through the transcriptome analysis (*fbn2*, *gal* and *gphb5*) and others previously determined to be related to skeletal physiology *(alp*, *bmp4*, *col1a1*, *col2a1*, *fgf23*, *igf1*, *mmp13*, *ocn*), were tested in the two independent sample sets. *Gphb5*, a recently discovered hormone, was significantly (*P* < 0.05) down-regulated in LJD affected fish in both sample sets, suggesting a possible hormonal involvement. *In-situ* hybridization detected *gphb5* expression in oral epithelium, teeth and skin of the lower jaw. *Col2a1* showed the same consistent significant (*P* < 0.05) down-regulation in LJD suggesting a possible cartilaginous impairment as a distinctive feature of the condition. Significant (*P* < 0.05) differential expression of other genes found in either one or the other sample set highlighted the possible effect of stage of development or condition progression on transcription and showed that anomalous bone development, likely driven by cartilage impairment, is more evident at larger fish sizes. The present study improved our understanding of LJD suggesting that a cartilage impairment likely underlies the condition and *col2a1* may be a marker. In addition, the involvement of *gphb5* urges further investigation of a hormonal role in LJD and skeletal physiology in general.

## Introduction

Lower jaw deformity (LJD) is a skeletal anomaly affecting the lower jaw of farmed Atlantic salmon (*Salmo salar* L.). Specifically, LJD is a downward curvature of the lower jaw involving dentary and glossohyal bones [1, 2]. LJD has been frequently observed and identified in both freshwater and seawater phases of production in all countries producing Atlantic salmon at/with different prevalence between years and populations [3–9]. Although LJD can occur in diploid populations at very low prevalence, LJD was linked to triploid Atlantic salmon in all recent studies cited above. In Tasmania (Australia) LJD prevalence of up to 30% has been reported in farmed triploid populations [7, 10]. Triploids are a valuable part of the annual harvest cycle as they do not undergo sexual maturation therefore can be harvested during the reproductive seasons providing fresh product all year round [7]. As a consequence, LJD affected triploid fish represent a considerable loss of production because they have lower growth rates and cannot be sold whole due to their visual unattractiveness [5, 7, 11]. Furthermore, fish affected by skeletal anomalies usually require hand-grading which is an expensive process and adds further cost [12, 13].

Although LJD is a frequently occurring skeletal anomaly in triploid Atlantic salmon its causes have not yet been investigated in depth. Only recently, development of LJD has been linked to dietary phosphorus (P) deficiency and a higher P requirement of triploid Atlantic salmon [8]. Nevertheless, the mechanisms underlying the onset of LJD are not known and could be multifactorial via a combination of genetic (triploidy and genetic background), nutritional (mineral or vitamin deficiency) and environmental (accelerated growth, low dissolved oxygen, elevated temperature and husbandry practices) factors [6–8, 14]. Although it seems to occur mostly during the freshwater phase, onset of LJD can occur at any time in development and prevalence and severity (i.e. worsening of the downward curvature) can increase over time [3, 4, 7–9, 11, 14, 15].

The lower jaw in Atlantic salmon is a heterogeneous organ constituted of different tissues. The bone of the lower jaw (i.e. dentary) is composed of compact bone directly ossifying around the Meckel’s cartilage [1, 16–18]. An assessment screening the differential gene expression between LJD and normal individuals, mostly focusing on cartilage and bone physiology, represents a basic approach to shed more light on the mechanisms underlying the condition. Obtaining a significant number of individuals affected by LJD, both in controlled experimental conditions and at commercial farm sites is difficult and labour-intensive. In this research, the opportunity was presented to analyse a sufficient number of fish affected by LJD from independent sample sets and at different developmental stages.

The aims of this study were to delineate for the first time through molecular techniques, transcriptome analysis and real-time qPCR, differential gene expression in the jaw of fish affected by LJD (compared to normal fish) in independent sample sets at different developmental stages and to detect genes which correlate with, and may characterise LJD, allowing the description of possible mechanisms underlying the condition. Furthermore, the specific gene expression pattern observed was used to propose the tissue responsible for the development of the condition. To support the analytical process, our findings are compared at a molecular level with anomalous skeletal processes described in other vertebrates.

## Results

### Transcriptome analysis of the experimental sample set

*De novo* assembly of the transcriptome data gave a total of 62,373 contigs (including scaffolded regions) with a minimum length of 500, a maximum of 14,769 and an average of 1,482 bases.

A total of 515 transcripts had at least 2 fold change between the LJD and Normal samples (unadjusted *P* < 0.05). When restricting RPKM ≥ 1 in at least one sample and defining the minimum as RPKM = 0.05, a total of 6,207 transcripts had at least 2 fold change between the LJD and Normal samples (unadjusted *P* < 0.05), indicating that most of the DGE can be attributed to transcripts which do not express in one group while they do in the other. Since in most cases these transcripts had very small RPKM values, we decided to focus on the 515 transcripts.

**Fig 1:**
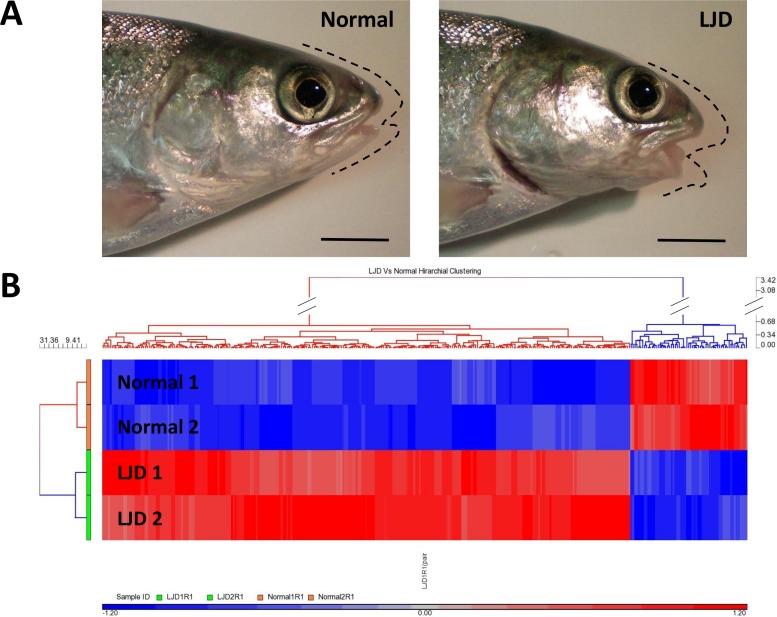
**a)** Atlantic salmon individuals displaying a normal jaw (Normal) on the left and a lower jaw deformity (LJD) on the right (scale bar = 1 cm). Dashed lines highlight normal and anomalous profiles. **b)** Hierarchical clustering showing differentially expressed transcripts between Normal vs. LJD grouped samples retrieved after a pairwise comparison. Each row corresponds to one sample tested and each column corresponds to a single differentially expressed transcript. On the hierarchical tree at the left side of the map, the upper half (orange) indicates the Normal samples and the lower half (green) indicates the LJD samples. Relative gene expression is indicated by colour: red is higher-level expression relative to the sample mean, blue is relatively lower-level expression, grey is no-change. The 452 genes on the left were up-regulated in LJD samples relative to the Normal samples. The 63 genes on the right were down-regulated in LJD samples relative to the Normal samples.

Out of the 515 transcripts, 452 were down-regulated and 63 were up-regulated in LJD. The hierarchical clustering of differential gene expression showed that samples clustered tightly together based on jaw trait (LJD/Normal; Fig 1B left), strengthening the validity of the differential gene expression analysis.

Following BLASTP selection according to E-value (for matches and hits) and RPKM-based filter (as described in the materials and methods section), two tables were produced: one consisting of 6 transcripts down-regulated in LJD samples (Table 1) and another consisting of 27 transcripts up-regulated in LJD samples (Table 2) ordered by E-value of the best hit (lowest to highest) (tables are shown in the following paragraphs).

**Table 1:** The six transcripts found down-regulated in LJD selected according to Sum of RPKM ≥ 5 across all 4 pooled samples ordered by E-value of the best hit (lowest to highest).

Transcript	Accession Best Hit	E-value Best hit	Accession Actual Hit	Predicted product [species]	E-value Actual Hit
**30200**	BAJ61837	0.00	BAJ61837	aggrecan [*Oncorhynchus keta*]	0.00
**8151**	CDQ60370	0.00	BAJ61837	aggrecan [*Oncorhynchus keta*]	0.00
**15298**	CDQ84852	1.61E-137	XP_005945737	cytosolic phospholipase A2 gamma-like isoform X2 [*Haplochromis burtoni*]	1.24E-89
**59181**	CDQ81732	7.53E-96	XP_005455982	glycoprotein hormone beta-5-like [*Oreochromis niloticus*]	2.20E-72
**662**	CDQ80483	5.85E-86	XP_007243147	protein FAM111A-like [*Astyanax mexicanus*]	6.29E-75
**26682**	CDQ61726	5.09E-82	XP_007242859	mannose-specific lectin-like [*Astyanax mexicanus*]	1.23E-62

**Table 2:** The 27 transcripts found up-regulated in LJD selected according to Sum of RPKM ≥ 5 across all 4 pooled samples ordered by E-value of the best hit (lowest to highest).

Transcript	Accession Best Hit	E-value Best Hit	Accession Actual Hit	Predicted product [species]	E-value Actual Hit
**3859**	CDQ85625	0.00	XP_006641632	IgGFc-binding protein-like [*Lepisosteus oculatus*]	5.60E-152
**718**	CDQ93202	0.00	XP_006641633	IgGFc-binding protein-like [*Lepisosteus oculatus*]	0.00
**407**	CDQ85625	0.00	XP_006641632	IgGFc-binding protein-like [*Lepisosteus oculatus*]	1.21E-151
**5789**	XP_005450984	0.00	XP_005450984	filamin-C-like isoform X3 [*Oreochromis niloticus*]	0.00
**1330**	CDQ86144	0.00	XP_008284200	xin actin-binding repeat-containing protein 1-like [*Stegastes partitus*]	0.00
**10204**	XP_007252649	0.00	XP_007252649	collagen alpha-2(VI) chain-like [*Astyanax mexicanus*]	0.00
**894**	XP_006808726	0.00	XP_006808726	von Willebrand factor A domain-containing protein 7-like [*Neolamprologus brichardi*]	0.00
**40854**	ACI68280	0.00	ACI68280	Four and a half LIM domains protein 2 [*Salmo salar*]	0.00
**26217**	ADD59862	0.00	ADD59862	immunoglobulin delta heavy chain constant region [*Salmo salar*]	0.00
**31731**	CDQ69338	0.00	XP_005163674	amylo-1, 6-glucosidase, 4-alpha-glucanotransferase isoform X1 [*Danio rerio*]	2.68E-128
**7575**	CDQ73728	4.69E-171	XP_008279287	pecanex-like protein 3 [*Stegastes partitus*]	5.79E-171
**22520**	CDQ71312	7.88E-158	XP_008293195	eukaryotic translation initiation factor 2-alpha kinase 4 [*Stegastes partitus*]	6.99E-120
**12561**	CDQ83306	3.64E-156	XP_006641633	IgGFc-binding protein-like [*Lepisosteus oculatus*]	1.42E-113
**41692**	CDQ61095	7.87E-156	XP_003966210	serum amyloid P-component-like [*Takifugu rubripes*]	2.85E-81
**5264**	ACI68585	2.30E-154	ACI68585	Heat shock protein 30 [*Salmo salar*]	2.30E-154
**15329**	NP_001118064	2.22E-135	NP_001118064	heat shock protein, alpha-crystallin-related, 1 [*Oncorhynchus mykiss*]	2.22E-135
**44416**	CDQ81901	3.22E-125	XP_007243338	protein NLRC3-like [*Astyanax mexicanus*]	1.85E-70
**15155**	CDQ66610	7.50E-124	XP_008292526	protein NLRC3-like [*Stegastes partitus*]	9.50E-121
**27480**	CDQ68760	3.73E-113	XP_008278793	sortilin-related receptor [*Stegastes partitus*]	1.33E-84
**2193**	CDQ61022	2.60E-108	ACO13356	Galactose-specific lectin [*Esox lucius*]	5.21E-09
**35957**	XP_007258003	3.18E-100	XP_007258003	ryanodine receptor 3-like [*Astyanax mexicanus*]	3.18E-100
**6273**	CDQ74429	1.20E-84	XP_008303180	fibrillin-2-like, partial [*Stegastes partitus*]	1.35E-82
**1876**	NP_001117045	2.06E-83	NP_001117045	cathelicidin antimicrobial peptide precursor [*Salmo salar*]	2.06E-83
**10290**	NP_001134766	3.32E-61	NP_001134766	Heat shock protein beta-7 [Salmo salar]	3.32E-61
**25466**	NP_001134309	3.03E-59	NP_001134309	Natterin-like protein [*Salmo salar*]	3.03E-59
**34091**	CDQ58999	2.04E-56	XP_003968600	serine/threonine-protein kinase Nek8-like [*Takifugu rubripes*]	3.05E-47
**45908**	ACY30362	6.08E-48	ACY30362	MHC class I antigen [*Salmo salar*]	6.08E-48

### Down-regulated transcripts

Among the 63 transcripts of this subset, 18 could be reliably annotated via NCBI database with E-value ≤ 1.00E^-40^. Of the 18 transcripts, six had a Sum of RPKM ≥ 5 across all four pooled samples (Table 1). Transcript 59181, which corresponded to glycoprotein hormone beta 5 (*gphb5*) had a much higher fold change (-4.3) between the groups compared to other transcripts. For five out of the above six transcripts, the best hit resulted in an unannotated product described for *Oncorhynchus mykiss* (rainbow trout), a closely-related Salmonid species. The actual hit for these transcripts resulted in products described in other Teleost species (Table 1). Transcript 30200, which corresponded to aggrecan isoform 1 *(acan1)* and that was annotated as aggrecan, had a best hit with a named product in the Salmonid *Oncorhynchus keta* (chum salmon) (Table 1).

### Up-regulated transcripts

Among the 452 transcripts of this subset, 176 could be reliably annotated via NCBI database with E-value ≤ 1.00E^-40^. Of the 176 transcripts, 27 had a Sum of RPKM ≥ 5 (Table 2). Transcript 40854 was annotated as Four and a half LIM domains protein 2, transcript 2193 corresponded to galactose-specific lectin *(gal)* and transcript 45908 was annotated as major histocompatibility complex (MHC) class I antigen. These transcripts had a much higher fold change (3.2, 3.5 and 4.6, respectively) between the groups, as compared with other transcripts. For 15 out of the 27 transcripts, the best hit resulted in an unannotated product described for the species *O*. *mykiss*. The actual hit for these transcripts resulted in products described in other Teleost species (Table 2). Seven of the remaining 12 transcripts had best hits with named products described for *S*. *salar*, and the last five with other Teleost species.

### Real-time qPCR validation

**Fig 2:**
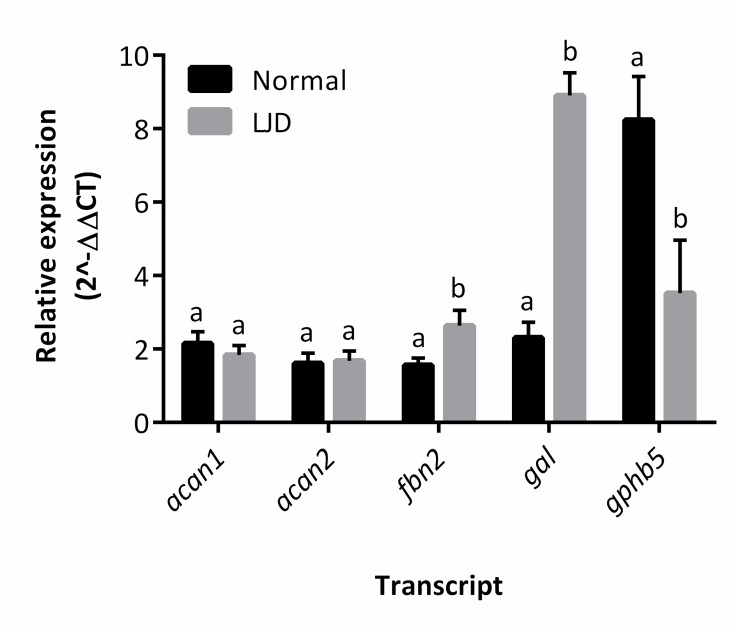
Relative expression (2^-ΔΔCT^, mean ± SEM) of selected transcripts (abbreviations described in the text) found to be differentially expressed in two different categories (Normal and affected by lower jaw deformity, LJD) after transcriptome analysis of triploid Atlantic salmon *Salmo salar* pre-smolts from the experimental sample set (*n* = 6 per jaw trait). For each transcript, expression level means were compared between different categories. Within each transcript, means significantly different from one another are indicated by different letters (*P* < 0.05).

The differential expression observed after the transcriptome analysis was confirmed for three out of five transcripts. In particular, fibrillin 2 (*fbn2*) and *gal* were significantly (*P* < 0.05) up-regulated in LJD affected fish while *gphb5* was significantly (*P* < 0.05) down-regulated in LJD affected fish (Fig 2). The differential expression of both *acan1* and *acan2*, that were significantly down-regulated in LJD affected fish according to the transcriptome analysis, was not confirmed by real-time qPCR assays (Fig 2).

### Real-time qPCR of different developmental stages

**Fig 3:**
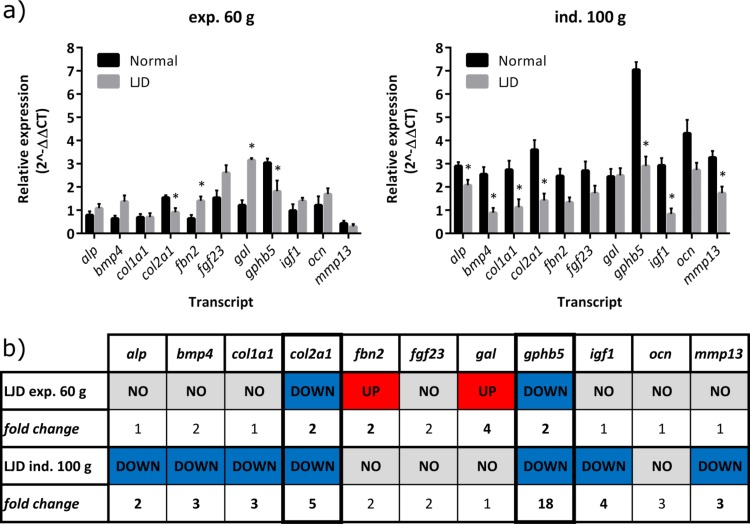
a) Relative expression (2^-ΔΔCT^, log-transformed mean + SEM) of the eleven transcripts tested in triploid Atlantic salmon *Salmo salar* individuals belonging to two independent sample sets, ‘experimental’ (exp. 60 g) on the left and ‘industrial’ (ind. 100 g) on the right, and displaying a normal lower jaw (Normal) or a lower jaw deformity (LJD) (*n* = 6 per jaw trait). Significantly different (*P* < 0.05) relative expression between traits is indicated by an asterisk. b) A graphic summary of the previous graphs to show significantly different regulation (UP—red, DOWN—blue, NO—grey) and corresponding approximate fold change (significant in bold) in LJD individuals only relative to Normal from the two independent sample sets. Columns of *col2a1* and *gphb5* have thicker borders to highlight consistent differential expression between independent sample sets.

Testing all the eleven transcripts available after initial selection and following transcriptome analysis in both the experimental and the industrial sample set, only *col2a1* and *gphb5* showed the same regulation pattern in both independent sample sets (Fig 3). In particular, *col2a1* and *gphb5* were significantly (*P* < 0.05) down-regulated in fish with LJD compared to Normal fish in the two independent sample sets (Fig 3). Other transcripts were found to be differentially expressed between traits in one sample set only. In particular, *fbn2* and *gal* were significantly (*P* < 0.05) up-regulated in LJD in the experimental sample set only while *alp*, *bmp4*, *col1a1*, *igf1* and *mmp13* were significantly (*P* < 0.05) down-regulated in LJD in the industrial sample set only (Fig 3). *Fgf23* and *ocn*, showed no differential expression between traits in both sample sets.

### Real-time qPCR for fish reared at different temperatures (14 vs 18°C)

**Fig 4:**
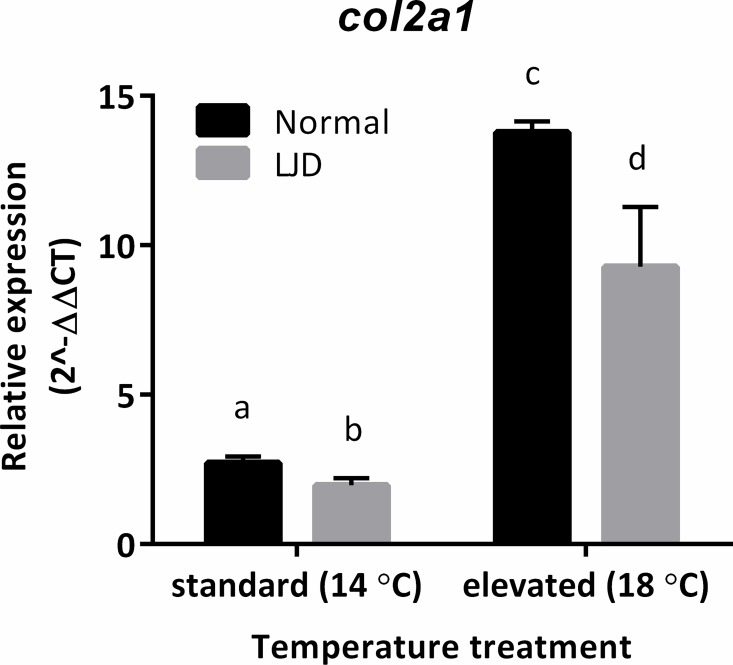
Relative expression (2^-ΔΔCT^, mean ± SEM) of *col2a1* in Normal and lower jaw deformity (LJD) affected triploid Atlantic salmon *Salmo salar* (*n* = 6 per jaw trait) from the experimental sample set at standard (14°C) and elevated (18°C) temperature. Means significantly different from one another are indicated by different letters (*P* < 0.05).

Among the transcripts tested for the effect of rearing temperature only *col2a1* showed to be differentially expressed. In particular, *col2a1* was significantly (*P* < 0.05) down-regulated in LJD within both temperature treatments and significantly (*P* < 0.05) up-regulated in both traits in the elevated treatment (18°C) compared to the standard treatment (14°C) (Fig 4).

### GPHB5 In-Situ Hybridization

**Fig 5:**
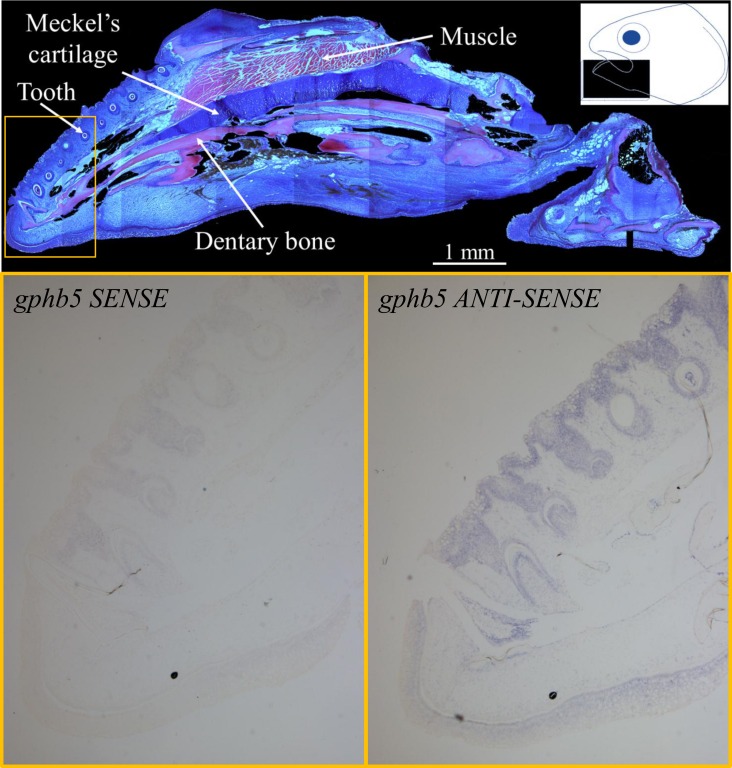
Top panel: a) a transverse H&E stained section of the lower jaw of a triploid Atlantic salmon (~60 g) affected by lower jaw deformity (LJD). Image series was tiled and black background was added using Photoshop. White arrows indicate the main anatomical features. In the top right corner the drawing illustrates the location and position of the sections obtained. Bottom panels: *in-situ* hybridization stained sections of a LJD affected jaw (region magnified from the corresponding H&E staining image is outlined in yellow at the top panel). b) Bottom left panel shows the negative control where *gphb5* sense probe was used while in c) the bottom right the tissue was hybridizing with the *gphb5* anti-sense probe. In the latter, the signal is detectable (purple stain) in the oral epithelium, around the teeth and in the skin at the tip of the jaw.

*In-situ* hybridization analysis showed the expression of *gphb5* in both traits (Normal and LJD) to be mostly in the skin and more evident in the oral epithelium and at the tip of the lower jaw (Fig 5). Furthermore, *gphb5* was also expressed around the teeth, in particular in the outer dental epithelium and around the dental papilla (Fig 5). Given the general low expression of the gene a quantitative differential expression between Normal and LJD was not observed.

## Discussion

In the present study, among the eleven genes tested in individuals displaying a normal lower jaw or a LJD, only *col2a1* and *gphb5* showed the same consistent pattern of differential expression, being down-regulated in LJD affected fish in both independent sample sets. This result implies that, in contrast to other genes found differentially expressed between traits either in one sample set or in another, *col2a1* and *gphb5* are reliable indicators of the mechanisms underlying LJD.

*Col2a1* is an exclusive marker of cartilage and is responsible for the expression of type II collagen, a basic protein for skeletogenesis in vertebrates [19–22]. In the present study, down-regulation of *col2a1* in LJD affected fish may thus indicate a compromised development of the Meckel’s cartilage through loss of structural integrity and incorrect growth trajectory (pointing downward) of the deriving or integrally-linked bone structure, the dentary bone (main lower jaw bone). Type II collagen is produced by chondrocytes and represents the most abundant protein in the cartilage extracellular matrix and is crucial for cartilage conformation and resistance [19, 22–24]. In Atlantic salmon, Meckel’s cartilage does not ossify and plays a crucial role in physically supporting the dentary, extending internally almost for its entire length [16–18]. Considering the consistency of down-regulation of *col2a1* in LJD-affected fish between independent sample sets from different developmental stages (60 vs 100 g), this finding suggests a cartilaginous impairment as a possible distinctive feature of the trait.

Defects in type II collagen have frequently been associated with the occurrence of impairments in cartilage and as a consequence bone development in humans and mice [25–36]. In support of our results, disruption of cartilage growth and development in LJD has been reported by previous studies. X-ray analysis showed a diminution of the Meckel’s cartilage in LJD-affected fish [2] and histological examination of LJD-affected Meckel’s cartilage showed different left to right thickness, implying an impaired development [1]. Likewise, the lower jaw of LJD-affected fish presented an incorrect assemblage and a smaller number of collagen fibres, adding to the evidence that the anomaly may be due to an impairment in the jaw cartilage development [37]. The studies mentioned above, analysed later stages of fish development compared to the current study, supporting our theory that cartilaginous impairment is a distinctive feature of LJD at any stage.

In the present study, the effect of water temperature on the up-regulation of *col2a1* in both jaw traits may be explained by an enhanced sensitivity of chondrocytes to higher temperatures (i.e. change in shape of chondrocytes) as shown for vertebral deformities in Atlantic salmon exposed to high water temperature [38]. In light of the above, prolonged exposure to high water temperature, in particular for Atlantic salmon above 14–15°C, may lead to a possible exacerbation of cartilage impairment and likely increased LJD severity in both originally normal and LJD affected fish, but with likely greater impact on the latter.

The consistent down-regulation of *gphb5* in LJD affected fish in both independent sample sets, as well as its localization through *in-situ* hybridization in this study, provides further insight into the onset of the condition, pointing towards a possible hormonal involvement. *Gphb5* is an evolutionarily well conserved glycoprotein hormone described for the first time in humans in 2002 [39]. Expression of *gphb5* occurs in several species in the pituitary, implying a role in hypothalamus-pituitary peripheral tissue (HPT) axis [39, 40]. *Gphb5* is known to activate the thyroid-stimulating hormone (TSH) receptors in cells of thyroid and it was named thyrostimulin due to its ability to stimulate thyrotropin receptors [40]. Nevertheless, the primary role of the hormone is still unknown [41]. *Gphb5* seems to have a paracrine rather than an endocrine function, implying that it can be expressed locally and induce changes in nearby cells [40, 41]. In the present study, the expression of *gphb5* in the skin cells is in accordance to that found in humans [42]. Furthermore, it has been recently shown that *gphb5* plays a paracrine role in skeletal development and bone formation [41]. In light of the above, our results may suggest that *gphb5* is expressed in the skin of the lower jaw and induce changes in cartilage or bone cells located at short distance. Furthermore, considering the aforementioned relation *gphb5*-thyroid, *gphb5* down-regulation and consequent lower expression in LJD-affected individuals may indicate an underactive thyroid. Underactive thyroid is generally linked with impairments in chondrocyte differentiation and linear growth as well as bone formation and mineralization [41]. Since, as suggested by our results, LJD could be the result of a cartilaginous impairment with resulting deleterious effects on bone development, the findings reported above support and reinforce our theory that *gphb5* is likely involved in mechanisms underlying LJD and, although not expressed specifically in cartilage or bone in the present study (mostly in oral epithelium, teeth and skin), *gphb5* may act through paracrine pathways. The literature for this relatively novel gene is limited and further investigations are needed to verify its role in skeletal development and HPT [40–42]. Nevertheless, we suggest that *gphb5* could be at least an indicator of a wider process behind the LJD condition in Atlantic salmon.

In support of a possible hormonal involvement in the condition, LJD has been recently linked to dietary phosphorus (P) deficiency during early stages of development in Atlantic salmon and different dietary P physiological requirements of triploids compared to diploids [8]. As bone in particular and the skeleton in general have a key role in endocrine regulation of minerals and nutrients [43], the onset of LJD could be either the result of a pre-existing impairment in the hormonal pathways of phosphate regulation, possibly involving *gphb5* as a thyrostimulin, or that different physiological P requirements, especially in triploids, and dietary P deficiency may trigger or facilitate LJD onset with the effect displayed in the differential expression of particular genes involved in phosphate hormonal control, with a possible role for *gphb5* as a thyrostimulin. In the present study, *fgf23* was not differentially expressed between traits in both/either sample sets. This suggests that while there was no differential expression of this important P homeostasis regulator at the fish development stages considered, an impairment in P homeostasis may have occurred earlier, or may occur later, in the progression of LJD. Alternatively, the impairment in P homeostasis linked to LJD in Atlantic salmon may occur through a different molecular pathway, not involving *fgf23*.

The predominant occurrence of LJD in triploids suggests that LJD it is likely to be associated with a disruption in timing/localization/function of gene expression, causing a temporal imbalance of the orchestrated function at the transcription or translation machinery required during embryonal development in order to generate complex traits. Nevertheless, the physiological differences resulting from triploidy (e.g. fewer and bigger cells and dietary P requirements) are likely interrelated in LJD occurrence [5, 8]. Triploid induction in Atlantic salmon is performed by pressure shocking eggs 30 min after fertilisation, during meiosis II [5, 44]. The shock suppresses cell division and prevents the extrusion of a polar body, resulting in cells having three sets of chromosomes instead of two [45]. Slight variations to pressure shock timing and temperature may confer developmental variability between individuals and an uneven triploidization per egg batch. This could result in either a lower triploidy induction rate [45] or potentially lead to imbalanced pathways where localised high levels of reactive oxygen species (ROS) occur and these then cause DNA breaks, as similar stresses (e.g. sonication) have been known to induce DNA breaks (http://cshprotocols.cshlp.org/content/2006/4/pdb.prot4538.short). Following the findings of the present study, *col2a1* and *gphb5* may be tested as early markers during embryonic development, in particular when Meckel’s cartilage differentiation takes place, to detect potential differential expression between triploids from the same batch and as a consequence to identify possible candidates that will develop LJD in future. In a recent publication, single-nucleotide polymorphism (SNP) in *col2a1* has been associated with mandibular prognathism, a lower jaw skeletal anomaly in humans [46]. Similar investigation should be undertaken for the role of *col2a1* in LJD in Atlantic salmon.

In the present study, additional genes important for skeletal physiology had different regulation patterns between independent sample sets (i.e. differential expression in one but not in the other sample set). Considering that the two sample sets included fish at different developmental stages, our results suggest that fish age and/or consequent progression of the condition may influence the expression of some genes relative to others. For instance, up-regulation of both *gal* and *fbn2* in LJD was detected only in the experimental sample set (the earlier developmental stage), which may be the result of a particular process occurring at that stage (due to development or condition). The process could be remodelling of the LJD affected jaw as a consequence of incorrect growth, likely driven by impaired Meckel’s cartilage development, or containment of the compromised structural integrity. In fact, members of the family of both galectins and fibrillins have been shown to be involved in cartilage and bone formation and development [47–54]. In particular, members of the galectins have been linked to diseases heavily affecting cartilage (i.e. rheumatoid arthritis and osteoarthritis) [55–57] and have a role in osteoblast differentiation, bone remodelling and osteoclastogenesis [49, 56, 58]. On the other hand, fibrillins play a crucial role in maintaining the integrity of connective tissues, correct formation and remodelling of extracellular matrix and bone structural development with effects on morphology and mechanical properties [53, 59].

In the industrial sample set, the LJD fish showed down-regulation of genes important for skeletal physiology, which may support the hypothesis of the progression of the LJD condition proposed above. Our results support a scenario where cartilage impairment leads to bone development impairment that becomes more evident at later stages. For instance, down-regulation of *igf1* in LJD may indicate impaired growth/development of cartilage and bone as well as poor mineralisation. *Igf1* is known to modulate skeletal development and in particular bone growth, resorption and mineralisation controlling proliferation and differentiation of chondrocytes, osteoblasts, osteocytes and osteoclasts [60–62]. Furthermore, given the possible interdependency of *igf1* and parathyroid hormone (PTH) for skeletal development [60, 63, 64] it remains unclear whether or not *igf1* down-regulation later in development and consistent down-regulation of *gphb5* in LJD in the present study may be part of the same cascade of impairment in bone hormonal control.

*Bmp4* down-regulation in LJD supports our hypothesis concerning the influence of developmental stage and/or condition progression on gene expression proposed above. Bone morphogenetic proteins (BMPs), which are multi-functional growth factors of the transforming growth factor b (TGFb) superfamily, play a fundamental role for cartilage and bone development and their normal functioning is required to avoid skeletal defects or malformations [65, 66]. In particular, *bmp4* has been repeatedly associated with mandibular or maxillofacial development and shaping in fish, birds and mammals [67–70], and has been linked before to oral malformations in birds [68, 71] and mammals [72], supporting and highlighting the possible significance of this gene in the LJD condition.

Finally, the remaining genes down-regulated in LJD-affected fish, *alp*, *col1a1* and *mmp13*, probably indicate that the bone is not growing/developing correctly as a consequence of cartilaginous impairment. In fact, all of these are well-known and important skeletal structural genes: *col1a1* encodes type I collagen, the main component of bone. *Alp* and *mmp13* are responsible for bone formation and mineralisation and cartilage and bone resorption, respectively [73–79]. In Atlantic salmon, all these genes have been already shown to be differential expressed in poorly mineralised and deformed vertebrae relative to normal vertebrae [8, 80, 81]. The current study suggests their involvement in the development of a skeletal anomaly affecting the lower jaw in Atlantic salmon.

In conclusion, although the causes of LJD are still unknown, we have made the first contribution, to our knowledge, to the understanding of the molecular mechanisms underlying the condition. We propose *col2a1* and *gphb5* as reliable candidates for detection of the condition due to their consistent down-regulation in LJD in two independent sample sets from two developmental stages. The down-regulation of *col2a1* here may indicate that LJD in Atlantic salmon is attributable to impaired development and structural defects of Meckel’s cartilage. In addition, we suggest that down-regulation and localization of *gphb5* infers a possible hormonal involvement in LJD. Although further investigation of the role of this hormone in LJD is needed, we have enhanced our understanding of a relatively novel hormone and showed for the first time that *gphb5* may be part of a mechanism behind a skeletal anomaly. Differential expression of other genes important for skeletal physiology in either one or the other sample set suggests that developmental stage or progression of the LJD condition could influence their transcription. Further molecular investigation of the marker candidates proposed in the current study is warranted. It is also recommended to carry out future annotation and analysis of paralogues using as reference the Atlantic salmon genome which has been recently made publicly available [82]. Finally, P homeostasis, skeletal hormonal control and mineralisation/structural characterisation of LJD in triploid Atlantic salmon require elucidation.

## Materials and Methods

### Sample background, selection and tissue source

Two independent sample sets of all-female triploid Atlantic salmon (*Salmo salar*) individuals were used in this study and both were provided by Petuna Seafoods hatchery in Cressy, Tasmania. All-female individuals were produced and triploidy was achieved as previously described [10, 44]. Briefly, shock to induce triploidy occurred 30 min after fertilisation when all-female eggs were subjected to a pressure at 9500 psi (655 Bar) for 4 min in water at 10°C, followed by 1 min for pressure release. Efficiency of the triploid induction was assessed by measuring erythrocyte nuclear length [83] and confirmed as 100% successful. The first sample set (defined “experimental” from now on) was collected at the end of the experiment described in [9]. Briefly, the individuals from the experimental sample set were triploized at hatchery site but reared from incubation up to the sampling event in experimental facilities. Individuals weighing approximately 60 g were sampled in March 2014 following exposure to a standard temperature treatment (14°C) for two months (additional samples were also collected from individuals exposed to an elevated temperature treatment of 18°C for two months for additional molecular investigations). The second sample set (defined “industrial” from now on) was reared at a hatchery site and collected in November 2015. Fish sampled weighed approximately 100 g and were subjected to different conditions compared to the experimental sample set (i.e. incubation and rearing temperature) and were derived from different broodstock. For each sample set, fish were euthanized by anaesthetic overdose (AQUI-S: 50 mg L^-1^) and the lower jaws of fish displaying LJD (LJD *n* = 6 per sample set) (Fig 1A) and of fish displaying a phenotypically normal jaw (Normal *n* = 6 per sample set) (Fig 1A) were dissected and placed in RNA preservation reagent (4 M ammonium sulphate, 25 mM sodium citrate, 10 mM EDTA; pH 5.2) to preserve RNA integrity. The samples were held at 4°C overnight and stored at– 20°C for a maximum of two months before processing for molecular analysis. For histological analysis, the lower jaws of individuals from the experimental sample set displaying LJD (*n* = 3) and phenotypically normal jaw (*n* = 3) were dissected and placed in Bouin’s solution overnight and then preserved in 70% ethanol for a maximum of four months before the analysis. All procedures were carried out with the approval of the University of Tasmania Animal Ethics Committee (approval number A0013044).

### RNA extraction and preparation for next generation sequencing

The lower jaw samples from the sample sets described above were carefully dissected, removing excess tissues in order to leave dentary bone and a thin layer of the surrounding tissues. Samples were then homogenized using a LabGEN 7 Series Homogenizer (Cole Parmer, Vernon Hills, IL, USA) in vials containing RNAzol® RT (Molecular Research Centre Inc., Cincinnati, OH, USA) for the isolation of total RNA, following manufacturer’s instructions. The isolated RNA was tested for quality and quantity using a NanoDrop 2000 spectrophotometer (Thermo Scientific, NanoDrop Products, Wilmington, DE, USA). For next generation sequencing, fish from the experimental sample set affected by LJD (*n* = 6) and phenotypically normal (Normal) (*n* = 6) were used. Equal amounts of RNA from three individuals from the same category were mixed to generate a total of four pooled samples representing two replicates of LJD and two replicates of Normal. The Agilent 2100 bioanalyzer (Agilent Technologies, Palo Alto, CA, USA) was used to validate quantity and quality of RNA. All samples had RIN (RNA Integrity Number) values higher than 7.

### Next generation sequencing and data handling

Samples were prepared for sequencing by the Australian Genome Research Facility (AGRF, Melbourne, Australia) according to the manufacturer’s instructions (Illumina, San Diego, CA, USA). Briefly, poly (A) mRNA was isolated using oligo (dT) beads and the addition of fragmentation buffer for shearing mRNA into short fragments (200–700 nt) prevented priming bias during the synthesis of cDNA using random hexamer-primers. The short fragments were further purified using QIAquick PCR purification kit (Qiagen, Hilden, Germany) and resolved with EB buffer for ligation with Illumina Paired-end adapters. This was followed by size selection (~200 bp), PCR amplification and Illumina sequencing using an Illumina Genome Analyzer (HighSeq 2000, Illumina, San Diego, CA, USA) performing 100 bp–paired end sequencing. The sequence reads were stored as FASTQ files. Overall, at least 4 Gb of cleaned data (at least 50 million reads) was generated for each of the four samples sequenced. Prior to assembly, quality of the FASTQ files was assessed using CLC Genomics Workbench v4 (CLC bio, Aarhus, Denmark), using default parameters. Based on the QC reports, FASTQ files were trimmed using CLC with default parameters with the addition of trimming 10 nucleotides from the 5’ of all reads.

### Bioinformatics and statistics

*De novo* assembly of the trimmed reads was performed in CLC Genomics Workbench v4 using default parameters with the exception of minimum contig length elevated to 500. Trimmed reads were mapped to the assembly in CLC Genomics Workbench v4 using default parameters with the exception of similarity fraction elevated to 0.9. BAM files (resulting in 77.05 ± 0.23% mapped reads per library) were then imported into Partek Genomics Suite (Partek Incorporated, St. Louis, MO, USA) for differential gene expression (DGE) analysis. All BAM files are available at https://dx.doi.org/10.6084/m9.figshare.4229555.v1. In Partek GS, categorical attributes were assigned to each duplicate in the LJD and Normal, followed by DGE analysis without restricting paired-end compatibility. One-way ANOVA was performed in Partek GS to compare reads per kilobase per million (RPKMs) with contrast between the LJD and Normal samples. The same one-way ANOVA procedure was performed following restricting RPKM ≥ 1 in at least one sample and defining the minimum as RPKM = 0.05. The final list of transcripts used in the analysis was retrieved by selecting transcripts having at least a 2 fold change between the LJD and Normal samples with an unadjusted significance level of *P* < 0.05.

The prediction of the amino-acid sequences corresponding to the transcripts was performed using the ORF-PREDICTOR website (http://proteomics.ysu.edu/tools/OrfPredictor.html). CLC main workbench 7.5 (CLC Inc, Aarhus, Denmark) at default parameters was used to perform a BLASTP (sequence comparisons and alignment) against the database of the National Center for Biotechnology Information (NCBI) and annotate and predict the most likely corresponding product (best hit) in Atlantic salmon. After the BLASTP, only matches with E-value ≤ 1.00E^-40^ were selected for the analysis. Discrimination of the best hit obtained for each sequence was performed based on the E-value ≤ 1.00E^-40^. In case the first best hit resulted in an unannotated product the second was chosen (named ‘actual hit’). In order to ascertain that the expression of the differentially expressed genes was not basal, only the subset of transcripts where the sum of RPKM in all four samples was ≥ 5, were considered.

### cDNA synthesis, probe and primer design for qPCR

Parallel to the transcriptome analysis, we tested in Normal and LJD samples from the experimental sample set a group of previously known transcripts whose function was annotated to be related to bone and cartilage physiology in vertebrates. The transcripts selected were: *alkaline phosphatase* (*alp*), *bone morphogenetic protein 4* (*bmp4*), *collagen type I alpha 1* (*col1a1*), *collagen type II alpha 1* (*col2a1*), *fibroblast growth factor 23* (*fgf23*), *insulin like growth factor 1* (*igf1*), *matrix metallopeptidase 13* (*mmp13*) and *osteocalcin* (*ocn*) (Table 3). Among the transcripts found to be differentially expressed after transcriptome analysis, the following were selected according to the quantitative values of differential expression, concurrently with their previously known or possible relation to bone and cartilage physiology: *fibrillin 2* (*fbn2*), *galactose-specific lectin* (*gal*) and *glycoprotein hormone beta 5* (*gphb5*) (Table 4). All the eleven transcripts reported above were later tested in Normal and LJD samples from the industrial sample set to compare gene expression patterns between the independent sample sets. Furthermore, some of the transcripts (*alp*, *col1a1*, *col2a1*, *mmp13* and *ocn*) were also tested in Normal and LJD samples from the additional sample set (elevated rearing temperature) in order to investigate the possible effect of the temperature on differential expression (14°C vs 18°C). 18S served as a housekeeping calibrator gene in both assays. Following RNA extraction and quantification, 1 μg of total RNA was reverse-transcribed into cDNA using Tetro cDNA synthesis kit (Bioline, London, UK), according to manufacturer’s instructions. Probes for qPCR were designed by the Universal ProbeLibrary System (Roche, http://www.roche-applied-science.com) and primers for transcripts previously annotated were purchased from GeneWorks Pty Ltd (Hindmarsh, SA, Australia) while primers for transcripts selected after transcriptome analysis were purchased from Sigma-Aldrich (Castle Hill, NSW, Australia).

**Table 3:** Primers and probes used for real-time qPCR designed from previously known transcripts whose function was annotated to be related to bone and cartilage physiology (abbreviations described in the text). * Sequence for *fgf23* is available in S1 File.

Transcript	Orientation	GenBank accession number	Tm	Sequence (5’-3’)	Probe cat.no.(Roche)
*alp*	Forward Reverse	FJ195609.1	59 59	cagctgagcagacagagtggcaacaaaggggaacttgtcc	04689011001
*bmp4*	Forward Reverse	NM_001139844.1	60 60	ggttgccgctaacactgacttggggtcttttcttagcgtct	04687582001
*col1a1*	Forward Reverse	FJ195608.1	60 59	agcctggtgctaagggagagccttagctccggtgtttcc	04688619001
*col2a1*	Forward Reverse	FJ195613.1	59 59	tcgacatgtctgccttcgtcagccctcatgtacctcaa	04693442001
*fgf23*	Forward Reverse	Sequence from our sequencing database*	59 60	ggatcagaagggtcaaccacaacacggtgccactggag	04685059001
*igf1*	Forward Reverse	EF432852.2	60 59	ggcttttatttcagtaaaccaaccgtccacaataccacggtta	04688546001
*mmp13*	Forward Reverse	NM_001140524.1	59 60	ccattccctcggtctcagaggtgctggggtttgtgtag	04684974001
*Ocn*	Forward Reverse	FJ172977.1	60 60	tgtgtgtgccactctattggatcttttctcactagcaggctttg	04689119001
*18s*	Forward Reverse	FJ710886.1	59 60	aggactccggttctattttgtgcggccgtccctcttaatc	04688546001

**Table 4:** Primers and probes used for real-time qPCR from selected transcripts found differentially expressed after transcriptome analysis in the experimental sample set (abbreviations described in the text). Sequences available in S1 File.

Transcript	Orientation	Tm	Sequence (5’-3’)	Probe cat.no.(Roche)
*fbn2*	Forward Reverse	59 60	cacgacagcgacacttgaaggctcacaactgtgacatgc	04687582001
*gal*	Forward Reverse	59 59	ctttgaactgcagtgagaccactcatgactcccatgatgacc	04694449001
*gphb5*	Forward Reverse	60 59	Tgtagggagggtcaaggacagagggcttcacatcaccac	04685059001

### Real-time qPCR assays

Real-time qPCR assays were performed in duplicates using FastStart Universal Probe Master (ROX) (Roche, Australia) according to manufacturer’s protocol in a Rotor-Gene 6000 Real-Time PCR Machine (Corbett Robotics Pty Ltd, Brisbane, Australia) with the following thermal cycling conditions: 95°C for 10 min, followed by 40 cycles of 95°C for 15 sec and 60°C for 60 sec. For each gene the control used was a duplicate no-template. Average cycle threshold for each duplicate was calibrated relative to 18S and basal expression levels (which refers to the lowest expressing tissue measured) and transformed to represent relative expression quantity as 2^-ΔΔCT^. Nonparametric test for independent samples (Mann-Whitney U test) was used to investigate significant differences between relative expression levels of each transcript between traits (Normal and LJD) and different temperature treatments. All data analyses were performed using GraphPad Prism version 6.00 for Windows (GraphPad Software, La Jolla, CA, USA) with a significance level of *P* < 0.05. Results are represented as mean ± standard error of the mean (SEM).

### General Histology

The left halves of the lower jaws collected from the experimental sample set were used for all histological analyses. Tissues were rinsed and decalcified for 72 h in a 10% EDTA solution buffered with 0.1 M TRIS base, pH 7.0 as described in [84] and supplemented with ProtectRNA™ RNase Inhibitor (Sigma-Aldrich, Castle Hill, NSW, Australia). After that, a protocol modified from [85] was used. Briefly, tissues were dehydrated gradually through a series of increasing alcohol concentrations and embedded in Paraplast® Plus (McCormick Scientific Leica™, North Ryde, NSW, Australia) according to conventional procedures. Serial sections of 7 μm were cut from the sagittal plane of the lower jaw, until reaching the area in which teeth, bone and Meckel’s cartilage were visible, and placed onto Superfrost™ Ultra Plus Adhesion Slides (Thermo Fisher Scientific, Scoresby, VIC, Australia). Duplicate and consecutive sections were used for Hematoxylin and Eosin (H&E) staining and *in-situ* hybridization.

### H&E staining

The slides were deparaffinized in xylene and rehydrated gradually through a series of decreasing alcohol concentrations (100%, 90%, 70%, 50%). After rinsing in water the slides were stained in hematoxylin for 4 min and rinsed again. Slides were placed for 30 sec in acidic alcohol (70% + 0.1% HCl), rinsed, stained in eosin for 3 min, rinsed again and gradually dehydrated through a series of increasing alcohol concentrations (50%, 70%, 90%, 100%), bathed in xylene and finally mounted.

### In-situ Hybridization

To design the primers for the *in-situ* hybridization probes, the sequences obtained from the transcriptome analysis of *gphb5* were blasted using Primer-BLAST (http://www.ncbi.nlm.nih.gov/tools/primer-blast/) and primers (Sequence 5’-3’—Forward gtgtacatggggtccacgtt and Reverse gagaagcctgtccttgaccc) purchased from Sigma-Aldrich (Castle Hill, NSW, Australia). Digoxigenin-labeled oligonucleotides for antisense and sense probes were synthesized using T7 RNA polymerase, and the probes were hydrolysed to reduce their length to approximately 200 bases, as described in the Digoxigenin Application Manual (Roche Applied Science, Indianapolis, IN). Slides with samples from the lower jaw were deparaffinised, rehydrated, rinsed in diethyl pyrocarbonate (DEPC)-treated water and then washed in PBS for 6 min. Samples were digested with 5 μg ml^-1^ of proteinase K (Roche Diagnostics GmbH) in PBS with Tween 20 (PBST) for 10 min at 37°C and incubated in PBST containing 2 mg ml^-1^ of glycine for 5 min at room temperature. Samples were rinsed again two times in PBST and fixed in 4% paraformaldehyde in PBS (10 mM phosphate buffer Na_2_HPO_4_, 150 mM NaCl, pH 7.4) for 4 min and again washed PBS containing 0.1% DEPC for 20 min and in PBS until pre-hybridization at room temperature. Pre-hybridization was performed at 48°C for 2 h in pre-hybridization buffer (50% 20X formamide, 10% saline sodium citrate, 40% dextran sulfate, tRNA 10 mg/ml, heparin 50 mg/ml and 10 mg ml^-1^ of sheared and denatured salmon sperm DNA). Hybridization was performed at 48°C overnight with 0.2 μg ml^-1^ of antisense and sense probes in hybridization buffer (identical to pre-hybridization buffer). Samples were washed three times for 5 min in 4X wash (50% formamide, 30% DEPC-treated water, 20% 20X SSC—0.15 M sodium chloride and 0.015 M sodium citrate—50 μl of Tween 20), three times for 5 min in 2X wash (50% formamide, 40% DEPC-treated water, 10% 20X SSC, 50 μl of Tween 20) and three times for 5 min in 1X wash (50% formamide, 45% DEPC-treated water, 5% 20X SSC, 50 μl of Tween 20) at 48°C. Samples were washed again three times for 5 min in 1X SSC with 0.1% Tween 20 and two times for 2 min in maleic acid buffer (MAB) (0.1 M maleic acid, 0.015 M NaCl, 0.1% Tween 20, pH 7.5) at room temperature. Blocking was performed at room temperature for 2 h with MAB block (2% BM block in MAB) and finally samples were incubated with Anti-Digoxigenin-AP (Roche, Australia) at 4°C overnight. Following incubation, samples were washed four times for 5 min with MAB, two times for 5 min with 1X alkaline phosphatase buffer (AP) (50% 1 M Tris, 40% DEPC-treated water, 10% 5 M NaCl, 0.01% Tween 20) and two times for 5 min 1X AP with 5% MgCl_2_ at room temperature. Final incubation was performed at 4°C overnight with 20 μl ml^-1^ of NBT/BCIP (Roche, Australia) in developmental buffer (5% polyvinyl alcohol in 1X AP with 5% MgCl_2_). Slides were dehydrated in alcohol 70%, 100% and cleared with xylene before mounting with DPX (Sigma-Aldrich). Sections were observed under a Nikon ECLIPSE E600 light microscope and photographed.cg

## Supporting Information

S1 File Sequences of selected transcripts. Sequences of *fbn2*, *gal* ad *gphb5* which were selected transcripts found differentially expressed after transcriptome analysis and of *fgf23*, a previously known transcript to be related to bone and cartilage physiology, whose sequence was retrieved during transcriptome analysis.
(PDF)
