# Supplementary material for: Multigenic Delineation of Lower Jaw Deformity in Triploid Atlantic Salmon (Salmo salar L.)
Source: PLoS One. 2016 Dec 15;11(12):e0168454. doi: 10.1371/journal.pone.0168454 (PMC5158070; doi:10.1371/journal.pone.0168454)
Supplement: S1 File — Sequences of fbn2, gal ad gphb5 which were selected transcripts found differentially expressed after transcriptome analysis and of fgf23, a previously known transcript to be related to bone and cartilage physiology, whose sequence was retrieved during transcriptome analysis. (PDF) [file pone.0168454.s001.pdf]

Sequences of *fbn2*, *gal* ad *gphb5* which were selected transcripts found differentially expressed after transcriptome analysis and of *fgf23*, a previously known transcript to be related to bone and cartilage physiology, whose sequence was retrieved during transcriptome analysis.

fbn2:

GTCTCCCACCCAGCCGTCACGACAGCGACACTTGAAGCTCCCGGGAACGTTGA
GACAGGCAGCATGCATGTCACAGTTGTGAGCCCCAATCTCACACTCATCGATA
TCTGTGCAGCCGGTGGAGCCCTTCTTAACAAAGTACCCCAGCTGGCAGTGACA
GATGAAAGATCCCTTGGTGTTCTCACAGTCTCCATGTAGACATATGTTAGGGTT
CAGCTCACACTCATTCACATCGATGCAGGTCCTCATGTCCATGGAGGCCATGA
ATCCATCGTAGCAGAGACAGCGGTACTCTCCAGGCACGTTGGTGCACTGGCCT
CCATCACAGATATCAGGGCTGTCCTCACACTCATCTATATCAGCGCAGGTCCTG
AGGTCAGGCATGAGAGCGTATCCCTCACTACAGCTGCATTCGTAGCTGCCCTCT
GAGTTGGTACAGTGTGTGTCACAGCCTCCGTTCATAATGGTACACTCATCAATA
TCCACA

fgf23:

AACATTTAATAAAGTGACTTCAGCTCAACTTACTTTTTTCTTCGAAGAAGACGC
ACAAATATTTCGGGTTTCAGCATGATTCTCATAAGAATCCATCCCGTAAACAGA
GTGCACTCATGTGCAGAATTTGCGTCCTAGAATTAAAAAGCACGGAAACCAAT
AGTTACATACTCACTAGCTACTGTGCTATAGTGAAAAATGAACAAAATACAAT
TTTCCGTCTTATGACTATATACAAATGACTAATATGCTCTAATAAGCCTGTCAC
GGTCAGAGTTTCACAGGTGTAAGAGTTGGGGTTTGTTTCCAGACCAGCTGGAA
AAGATCCAACGTGAACGCGCACACAACTTATATTCTTAAAACCAGGTGTACAA
CGGCAAGTGTCCTGTCCTCTTATCCCACCATTGCATTCATGATACGGGGACTGG
GAGGGGGGTTTAGGGAATGCACGTTCCACGGGTCGTCGGAGGGAGACTGAGG
GGTCTCCCGGGAGATGTTGCGTCCCTCGGGGAGTTCAGCCTCCTGGTCCATCTC
CAAGGTGTCATCCAGCTCCGGCATAGCCCGGGAGTCCTCATCCTCCTCCGTTTG
ACCCAGCATGTTGTACGGATCAGAAGGGTCAACCACCCGGTTTCTCTTCTCCCG
GTGCAAGAGGCGCTCCAGTGGCACCGTGTTCTTCTCCGACAGGAAGAGGGAGG
TTTGCGGTAGATTCTGGCCCACAGTGTACACTTGCTTTATTCCTTCCAAGTTGA
GCAGAATACCAGTTCTGCAAGAGTAGTACACGTCGCGGTGGTTTTCCAATAAC
TTGTGGTTAAAAAGACAGTCTTCCTTGTGGCAAACGG

gal:

CTTTGAACTGCAGTGAGACCACCAGAGCACCACCATTGTCCAGGTCATCATGG
GAGTCATGACTGTGTCTGTTCTTTTCTGCGTCCTCCTCACCTTCCTGGGGACCGA
GGTGACTGGGAGGTCTCCCCCATGGTATCCCAAATTACCCCCACCAGTGGAGA
GATTTGCAAGAAGATCTGAACCAATACCAGATCCGTACCAGTGTCCATCTGGG
TTTTCTCGACCCAACCGTTACTGGAGCACTTGCTACCGTTTCGACAGCAGTTTG
AAGTCAATGTCGGCGGGAAAGAATGCATGCCGTCAAAGAGGTGGATCCCTTGC
TTCTATCACCAACAGTAACGTGTACGAGTATGCTCTTTCCCTGGTAGAAGATGG
ATATATCTGGGTTAAGGGGAGCCATGCGTCTGAATTTCCCCAGAAGAACTGGT
TGAAAGGCTCTGTGCGTGACCAGAATGGAAAGCATGAGGGCTGCTTGACATTG
GTGAAATTTGGTGAAAAAACTGGTTTTGATGATATCAGCTGTTCCAGTATGGAC
AACACCGCATTCACTCTCTGCGAGATAGAAACGTCTTAATTCTTAGTTTAAGAA
GGCCAAACAGAGAGTGGATTTTGCCAATACTCTCTGATTTTCTTTTTCTACTAT
CTCACTAAATTGTTCTGAATTTACTTTAAGTCTAATACTTAGAGACAATATAAT

GAAAAGGGATGTAATGGCTACATATTCATACATAATGATACATTGATTCAATT<br>GCATTTCACGTGGCCTTTTTTCGACTGAAAATAATAGTCTGGTTTTGCAATGGA<br>ATTGTCACTGTAGTATTCTGGAAGCTATCTTTAAGATACTGTACCAAACATGTG<br>GGTACGTACAATCCCATTTCAAGATGAACTATATAATTACGAACACATACAGT<br>TATTCTCAATACTTACTCTAACAGTGGAAATAGGCTATACCCCATTATATCTGG<br>AATATCTATTGAATTTGTGGAATTGTTGTGTCATGTACATCCCTCCTTGCTTACC<br>TTGCATATCTTTTCGAATAAAAACATTCAGCATCAAAAAAAAAAGATCGGAAG<br>AGCACACG
gphb5:<br>AAAGGTGATTTCCATTCTCTCCATAGAGTAAACATCCAGCGCTGCTGCTCTCCC<br>TGTCCAGTGCCAGTGATGGTGCTGCAGTCAGACAGAGGTGATACACTCAGTGG<br>TACTGGTCAGGCACACTCCACAGTCACACCTCAGGGCTACAGGGTAGGTGTAC<br>ATGGGGTCCACGTTGGCTGAGCAGTTAGGCAGCCGCACCGTCACCAGGCGGGT<br>CTCGTTGTAGGTACACACACGCTGGTATGACTCTATGTAGGGAGGGTCAAGGA<br>CAGGCTTCTCCCAGGTCTCACAACGCCCCCAGCAGGCGTCGGTGGTGATGTGA<br>AGCCCTCCACAGCCTGGTTTCCTGGCCAGGAAGGTGAACTCCCGGACGGCACA<br>GCCAATGAAGCGGCGCAGGTTGACGGCTGAAGCCTGACTCAGTGTCTCTGGGT<br>GAAGCCATGACCACAGCCCTGTACAGCACAGCAGCACAGCACACCACCACTG<br>AGTACCTCTCCTCTGGGTCATCTCACAACTGGTCTCACTGCAGTGGTTCTCCAC<br>TGGAGAAAACTGCCACTACTGAACCTCTGAATCAGGAGCTACAGTAACTCCAG<br>TAGCTGTGATGTCTCTGACAGCAGAATCACCCGATAA
